# Invasive *Salmonella* Typhimurium ST313 with Naturally Attenuated Flagellin Elicits Reduced Inflammation and Replicates within Macrophages

**DOI:** 10.1371/journal.pntd.0003394

**Published:** 2015-01-08

**Authors:** Girish Ramachandran, Darren J. Perkins, Patrick J. Schmidlein, Mohan E. Tulapurkar, Sharon M. Tennant

**Affiliations:** 1 Center for Vaccine Development, University of Maryland School of Medicine, Baltimore, Maryland, United States of America; 2 Department of Medicine, University of Maryland School of Medicine, Baltimore, Maryland, United States of America; 3 Department of Microbiology and Immunology, University of Maryland School of Medicine, Baltimore, Maryland, United States of America; 4 Division of Pulmonary and Critical Care, University of Maryland School of Medicine, Baltimore, Maryland, United States of America; Oxford University Clinical Research Unit, Viet Nam

## Abstract

Invasive non-typhoidal *Salmonella* (iNTS) are an important cause of septicemia in children under the age of five years in sub-Saharan Africa. A novel genotype of *Salmonella enterica subsp. enterica* serovar Typhimurium (multi-locus sequence type [ST] 313) circulating in this geographic region is genetically different to from *S*. Typhimurium ST19 strains that are common throughout the rest of the world. *S*. Typhimurium ST313 strains have acquired pseudogenes and genetic deletions and appear to be evolving to become more like the typhoidal serovars *S*. Typhi and *S*. Paratyphi A. Epidemiological and clinical data show that *S*. Typhimurium ST313 strains are clinically associated with invasive systemic disease (bacteremia, septicemia, meningitis) rather than with gastroenteritis. The current work summarizes investigations of the broad hypothesis that *S*. Typhimurium ST313 isolates from Mali, West Africa, will behave differently from ST19 isolates in various *in vitro* assays. Here, we show that strains of the ST313 genotype are phagocytosed more efficiently and are highly resistant to killing by macrophage cell lines and primary mouse and human macrophages compared to ST19 strains. *S*. Typhimurium ST313 strains survived and replicated within different macrophages. Infection of macrophages with *S*. Typhimurium ST19 strains resulted in increased apoptosis and higher production of proinflammatory cytokines, as measured by gene expression and protein production, compared to *S*. Typhimurium ST313 strains. This difference in proinflammatory cytokine production and cell death between *S*. Typhimurium ST19 and ST313 strains could be explained, in part, by an increased production of flagellin by ST19 strains. These observations provide further evidence that *S*. Typhimurium ST313 strains are phenotypically different to ST19 strains and instead share similar pathogenic characteristics with typhoidal *Salmonella* serovars.

## Author Summary

Non-typhoidal *Salmonella*, such as *Salmonella* Typhimurium, generally cause self-limiting gastroenteritis. However, in sub-Saharan Africa, a novel genotype called sequence type (ST) 313 is circulating and causes bloodstream infections in infants and HIV-infected individuals. In contrast, the most common genotype found throughout the rest of the world is ST19, which has been highly studied. Currently, the pathogenesis of *S*. Typhimurium ST313 is not well understood. In our study, we present evidence that *S*. Typhimurium ST313 strains from Mali, West Africa, survive and replicate better within a diverse set of primary human and murine macrophages and cell lines, inducing significantly less host-cell death as compared to *S*. Typhimurium ST19. Flagellin expression in the *S*. Typhimurium ST313 strains was found to be attenuated compared to *S*. Typhimurium ST19, thereby causing decreased inflammation. We conclude that *S*. Typhimurium ST313 have evolved phenotypically to be an intermediate between *S*. Typhimurium ST19 and the highly invasive typhoidal *Salmonella* serovars *S*. Typhi and *S*. Paratyphi A.

## Introduction

Invasive non-typhoidal *Salmonella* (iNTS) are increasingly being recognized as an important cause of septicemia and meningitis in sub-Saharan Africa, especially in children under the age of 5 years [1]. Individuals at high risk of developing iNTS infections in Africa include young children (particularly with severe anemia from malaria) or with hemoglobinopathies and adults with untreated HIV infections [2], [3]. Annual incidence rates of 200–350 cases of iNTS infections/10^5^ infections in infants and toddlers have been recorded. A 20–25% case fatality rate has been observed in children and up to 50% case fatality in adults with HIV [4], [5]. Interestingly, only ∼30% of children who present with iNTS also present with gastroenteritis [6].

The majority of iNTS in sub-Saharan Africa belong to *Salmonella enterica subsp. enterica* serovars Typhimurium and Enteritidis [1]. Multi-locus sequence typing (MLST) performed on *S*. Typhimurium strains isolated from blood of children in Kenya and Malawi revealed that a novel sequence type, ST313, is circulating in sub-Saharan Africa [4]. In comparison, the sequenced *S*. Typhimurium strains LT2, SL1344, and NCTC13348 are ST19 and are associated with classical gastroenteritis. The ST19 genotype is the most common sequence type of *S*. Typhimurium found throughout the world [7]. Genomic analysis of an invasive *S*. Typhimurium ST313 clinical isolate, strain D23580 from Malawi, has revealed that compared to the sequenced *S*. Typhimurium ST19 strains, this isolate has undergone genomic degradation, acquired several pseudogenes and a large number of gene deletions in the chromosome [4]. This phenomenon has previously been observed in *Salmonella* Typhi and *Salmonella* Gallinarum as a means for host adaptation [8]. Some of the pseudogenes of *S*. Typhimurium D23580 are homologous to those that exist in the typhoidal servoars *S*. Typhi and *S*. Paratyphi A, which cause enteric fever [9], [10], [11]. Sequencing of *S*. Typhimurium strains isolated from Malawi, Mali, Kenya and Nigeria revealed that there are two closely related phylogenetic lineages of *S*. Typhimurium ST313 and that lineage I isolates have recently been replaced by those from lineage II that harbor a chloramphenicol acetyl transferase gene on a Tn*21* element, due to the use of chloramphenicol for the treatment of iNTS disease [12].

*Salmonella,* acquired following ingestion of contaminated food or water, must transit throughout the gastric acid barrier to reach the small intestine where they target M cells overlying gut-associated lymphoid tissue [13]. The bacteria are engulfed by intestinal macrophages and enter the lymph drainage allowing them to reach the blood circulation via the thoracic duct and to be taken up by the organs of the mononuclear phagocyte system (previously called the reticuloendothelial system) including the liver, spleen and bone marrow. When ingested by macrophages or upon invading enterocytes, *Salmonella* induce membrane ruffling [14]. Within macrophages they survive and replicate within *Salmonella*-containing vacuoles [15], [16], [17].

Accumulated data thus suggest that *S*. Typhimurium ST313 strains circulating in sub-Saharan Africa are genetically different from *S*. Typhimurium ST19 strains and have acquired features in common with typhoidal serovars causing enteric fever. We therefore hypothesize that *S*. Typhimurium ST313 strains are also phenotypically different from *S*. Typhimurium ST19 strains and share pathogenic mechanisms with typhoidal *Salmonella* serovars. Here, we examined the interaction of invasive *S*. Typhimurium ST313 and ST19 strains from Mali, West Africa, with macrophages, an important step in the pathogenesis of these bacteria.

## Materials and Methods

### Ethics statement

Neutrophils were obtained from the peripheral blood of healthy human donors under a protocol approved by the University of Maryland, School of Medicine Institutional Review Board. All healthy blood donors provided their written informed consent. Mouse experiments were carried out in strict accordance with the recommendations in the Guide for the Care and Use of Laboratory Animals of the National Institutes of Health. All protocols (Protocol number: 0212016) were reviewed and approved by the Animal Care and Use Committee at the University of Maryland, School of Medicine.

### Bacterial strains, plasmids and growth conditions

The bacterial strains used in this study are shown in Table 1. All *Salmonella* strains were grown to log phase (OD_600_ = 1.5) in animal-product free HY-Soy (HS) media (0.5% Hy-yeast [Sigma], 1% Soytone [TEKNova]) containing 0.3 M NaCl at 37°C unless otherwise indicated. *S*. Typhimurium I77 (ST19) and D65 (ST313) were labeled with green fluorescent protein (GFP) by electroporation of plasmid pGEN206 which harbors *gfpuv* expressed by the OmpC promoter [18]. All *guaBA* mutants were grown in medium containing 0.005% (wt/vol) guanine. Motility tests were performed as previously described [19].

**Table 1:** Bacterial strains used in this study.

Serovar	Sequence type	Strain	Source/characteristics	Reference
Typhimurium	19	I77	Blood culture, Mali	[46], [47]
		I77 (pGEN206)	GFP-expressing I77	This study
		I41	Blood culture, Mali	[46], [47]
		I89	Blood culture, Mali	[46], [47]
		S52	Blood culture, Mali	[46], [47]
		SL1344	Sequenced reference strain	[48]
	313	D65	Blood culture, Mali	[46], [47]
		D65 (pGEN206)	GFP-expressing D65	This study
		Q55	Blood culture, Mali	[46], [47]
		S11	Blood culture, Mali	[46], [47]
		S12	Blood culture, Mali	[46], [47]
		A13	Blood culture, Mali	[46], [47]
		D70	Blood culture, Mali	[46], [47]
		S42	Blood culture, Mali	[46], [47]
		P142	Blood culture, Mali	[46], [47]
		P104	Blood culture, Mali	[46], [47]
		Q65	Blood culture, Mali	[46], [47]
		R86	Blood culture, Mali	[46], [47]
		S72	Blood culture, Mali	[46], [47]
		D23580	Blood culture, Malawi	[4]
		CVD 1930	*S*. Typhimurium D65 Δ*guaBA*	Tennant SM, Schmidlein PJ, Simon R, Pasetti MF, Galen JE and Levine MM, Unpublished data
		CVD 1931	*S*. Typhimurium D65 Δ*guaBA* Δ*clpX*	Tennant SM, Schmidlein PJ, Simon R, Pasetti MF, Galen JE and Levine MM, Unpublished data
Typhi		Ty2	Wild-type reference strain	[49]
Paratyphi A		ATCC9150	Wild-type reference strain	American Type Culture Collection, Manassas VA

### Harvesting peritoneal macrophages from mice

Specific pathogen-free six-to eight-week-old female BALB/c and CD-1 mice were purchased from Charles River Laboratories (Wilmington, MA). CD-1 and BALB/c mouse macrophages were obtained by injecting mice intra-peritoneally (i.p.) with 3 ml of thioglycollate medium (Sigma-Aldrich). Two days later, mice were euthanized and peritoneal macrophages were harvested by injecting 5 ml of PBS into the peritoneum and flushing out the cells. The macrophages were suspended in complete RPMI1640 medium (Invitrogen) supplemented with 10% (v/v) fetal bovine serum (FBS), 100 U/ml penicillin, 100 µg/ml streptomycin and 1 mM sodium pyruvate and allowed to incubate overnight at 37°C with 5% CO_2_.

### Isolation of human peripheral blood mononuclear cells (PBMCs)

Human PBMCs were isolated from whole blood by Ficoll separation. Briefly, blood was collected in tubes containing an equal volume of 2% (v/v) dextran in normal saline with 25 mM sodium citrate. The tubes were allowed to rest for 30 min at RT to cause sedimentation of the majority of the red blood cells to the bottom. The pale orange top layer containing the white blood cells were collected and carefully layered over a 50% volume of Ficoll-Hypaque (Sigma-Aldrich). The tubes were centrifuged at 400×*g* for 30 min at 20°C. The buffy coat that formed in the middle of the gradient was collected. These cells were washed three times in PBS and RPMI1640 media and incubated at 37°C with 5% CO_2_ overnight.

### Uptake by J774 macrophages

Mouse macrophage J774 cells were maintained using DMEM containing 4.5 g/l D-glucose, 4 mM L-glutamine and 1.5 g/l sodium bicarbonate (Corning Cellgro) supplemented with 10% [v/v] FBS. 24-well plates were seeded with 4×10^5^ cells/well and incubated for 1 day. Semi-confluent J774 cell monolayers were washed twice with sterile PBS which was replaced with fresh tissue culture medium. Bacteria were prepared by performing a 1∶100 dilution of an overnight culture in fresh HS media containing 0.3 M NaCl and incubating at 37°C until the OD_600_ reached 1.5. A 10 µl aliquot of bacterial cell suspension (4×10^7^ CFU (colony forming units)) was added to a semi-confluent layer of J774 cells. The plate was centrifuged at 800×*g* for 10 min at 22°C to bring bacteria into contact with the cells. The plate was then incubated at 37°C in a 5% CO_2_ incubator for 30 min. The cells were washed three times with PBS, replaced with fresh medium containing 100 µg/ml gentamicin (which kills extracellular bacteria only), and the cells incubated for 1 hour at 37°C in humidified atmospheric air containing 5% CO_2_. The medium was then removed and the cells were washed three times with PBS. Finally, the J774 cells were lysed by vigorous pipetting in 200 µl of 1% (v/v) Triton X-100 (t-Octylphenoxypolyethoxyethanol). Intracellular bacteria were enumerated by performing viable counts. Each strain was tested in duplicate wells and on at least three separate occasions. The percentage of bacterial cells taken up by J774 cells (i.e., percent phagocytosis or uptake) was calculated by dividing the number of bacteria that survive the gentamicin treatment by the inoculum size and multiplying by 100.

### Survival in macrophages

THP-1 and U937 cells were grown in complete RPMI1640 medium and activated with 10^−8 ^M PMA (phorbol 12-myristate 13-acetate) for 24 h. 4×10^5^ cells were seeded into each well of a 24-well plate and incubated at 37°C for 24 h in a CO_2_ (5%) incubator. Bacterial strains were inoculated in HS media containing 0.3 M NaCl and incubated under aerobic conditions at 37°C until the OD_600_ reached 1.5. Activated THP-1 and U937 macrophages and untreated murine peritoneal macrophages and PBMC's were infected at a MOI (multiplicity of infection) of 10∶1 (bacteria: cells). Each bacterial strain was used to infect four wells of macrophage cells. The trays were centrifuged for 10 min at 400×*g* at room temperature to bring the bacteria in contact with the cells. The cells were incubated for 30 min at 37°C in a 5% CO_2_ incubator following which they were washed three times with PBS. RPMI1640 containing 100 µg/ml gentamicin was added to the cells and incubated for a further 30 min. For one pair of cell aliquots, media was replaced with fresh RPMI1640 containing 5 µg/ml gentamicin and 10^−8^ M PMA and incubated at 37°C for 24 h. The other set of cells were lysed using 200 µl of 1% Triton X-100. The cells were serially diluted in media and plated on HS agar to determine the number of intracellular bacteria at t = 0. Lysis and viable counts were repeated for the cells that were incubated for 3, 8 and 24 h to determine the intracellular survival of the strains. Each strain was tested in duplicate wells and on at least three separate occasions. Results were expressed as % intracellular survival which was defined as the CFU at t = 0 divided by the CFU at t = 3 h, 8 h or 24 h times 100.

### Confocal microscopy

PMA (10^−8^ M)-activated THP-1 cells were seeded at a density of 2–7×10^5^ cells/well in an 8-well chamber slide (Nunc) and allowed to adhere overnight at 37°C in a 5% CO_2_ incubator. The cells were infected with GFP-expressing *S*. Typhimurium I77 (pGEN206) and D65 (pGEN206) strains at a MOI of 10∶1 (bacteria: cells) for 30 min at 37°C in a 5% CO_2_ incubator following which they were washed three times with PBS and incubated for 30 min in RPMI1640 containing 100 µg/ml gentamicin. Cells were replaced with fresh media without gentamicin and incubated for 24 h. Cells were fixed for 8 min at room temperature with 4% v/v paraformaldehyde (USB Chemicals). The cells were then washed three times with PBS and blocked for 30 min in blocking buffer (1X PBS containing 0.03% [v/v] Triton X-100, 1% [w/v] BSA and 1% [v/v] donkey serum [Invitrogen]). The cells were washed three times for 5 min with washing buffer and incubated for 8 min with Acti-stain phalloidin (Red) (Life Technologies) or anti-human LAMP-1 (Cell Signaling) at a 1∶100 dilution, followed by washing and incubating for 5 min at room temperature with 5 nM Hoechst (Pierce) in PBS. The samples were washed three times for 5 min each with PBS and mounted using Vectashield mounting medium (Vector Laboratories). The samples were visualized using an Olympus confocal microscope (Model BX61) equipped with FluoView software. The number of bacteria per infected cell was determined by counting at least 12 cells in six different regions of the slide. The number of cells infected with *S*. Typhimurium I77 (ST19) and D65 (ST313) per field was determined by viewing cells in 12 different fields of the slide.

### Cytotoxicity assay to determine apoptosis

*S*. Typhimurium I77 (ST19) and D65 (ST313) were grown to an OD_600_ of 1.5. THP-1 cells were either left uninfected or infected with *S*. Typhimurium strains at a MOI of 10∶1. The cells were harvested by trypsin treatment at 24 h p.i. and the number of viable, mid-apoptotic and apoptotic cells was determined using Guava ViaCount (Millipore) according to the manufacturer's protocol. The Guava ViaCount reagent is used to determine cell count and viability based on differential permeability of two DNA-binding dyes. Viability was determined using the CytoSoft software containing the ViaCount module.

### Western immunoblot analysis

Whole cell lysates from infected, differentiated THP-1 cells were obtained by the addition of lysis buffer (20 mM HEPES, 1.0% [v/v] Triton X-100, 0.1% [w/v] SDS, 150 mM NaCl, 10 mM NaF, 1 mM phenylmethylsulfonyl fluoride) and subsequent incubation at 4°C. Cell lysates were separated by electrophoresis in a denaturing 12% SDS-PAGE, and subsequent transfer to PVDF membrane. Blots were incubated overnight with relevant primary antibodies at 4°C, washed 3 times with PBS, and then incubated with appropriate HRP-conjugated, secondary antibody (Jackson Immunochemicals). Blots were developed following incubation in ECL Plus Western Blotting Detection Reagent (Amersham Bioscience). Antibodies against phospho-p65 (serine 536), total p65, and total p38 were obtained from Cell Signaling. Rabbit polyclonal antibody against phospho-p38 was obtained from Promega.

For analysis of flagellin expression by *S*. Typhimurium I77 and D65, bacterial lysate and supernatant were separated on 4–15% gradient SDS-PAGE (Biorad) and transferred to PVDF membrane. The blot was incubated overnight in a 1∶1000 dilution of FliC monoclonal *S*. Typhimurium (IE6) AH12 antibody and a goat-anti-mouse secondary antibody (Cell Signaling). Densitometry analysis of the bands was performed using the ImageJ software (http://imagej.nih.gov/ij/).

### Infection of HEK293-Luc cells and luciferase assays

Human epithelial kidney cells (kindly provided by Dr. Raphael Simon) stably expressing the firefly luciferase reporter under control of an NF-κB-dependent promoter (HEK293-Luc) [20] were maintained in DMEM (Invitrogen) supplemented with 10% (v/v) FBS and 1 mM sodium pyruvate. Growth was at 37°C in a 5% CO_2_ atmosphere; monolayers of cells in 24-well plates were used for NF-κB activation analyses. Monolayers of HEK293-Luc cells were treated for 4 h with live or heat-killed bacteria (1×10^5^ CFU) or supernatant as indicated. Extracts were prepared and luciferase activity was measured according to the manufacturer's protocol (Promega) using a LMAX II^384^ (Molecular Devices).

### Cytokine expression analysis by quantitative real-time PCR (qRT-PCR)

Total mRNA was isolated from infected THP-1 cells using TRIZOL (Invitrogen) reagent according to the manufacturer's instructions. A total of 1 µg of RNA was utilized in oligo(dT) cDNA synthesis (Promega RT system A3500). qRT-PCR was carried out using an ABI Prism 7900HT Sequence Detection System (Applied Biosystems) utilizing SYBR Green Reagent (Applied Biosystems) and transcript-specific primers [21]. mRNA expression profiles were normalized to levels of the housekeeping gene hypoxanthine-guanine phosphoribosyltransferase (HPRT) in each sample and the fold change in expression was calculated by the 2^−ΔΔCt^ method [22].

### Cytokine protein analysis

Cytokine levels of TNF-α, IL-1β, INF-γ, IL-4 and IL-8 in supernatants of THP-1 cells infected with ST19 and ST313 at 3 h and 8 h p.i. were assayed by the cytokine core facility at the University of Maryland School of Medicine using a multiplex immunoassay, with standard curves (Quansys Biosciences).

### Statistical analysis

Student's *t-*test was performed using Prism software (GraphPad Software, San Diego, CA). A two-tailed P value of <0.05 was taken to indicate statistical significance.

## Results

### *S*. Typhimurium ST313 strains are phagocytosed more efficiently by murine J774 macrophages and have increased survival within human macrophages compared to *S*. Typhimurium ST19 strains

We evaluated the ability of *S*. Typhimurium ST19 and ST313 strains isolated from the blood of infants and children in Mali, West Africa, to be phagocytosed and to survive within various macrophage cell lines. We tested up to 4 invasive *S*. Typhimurium ST19 strains and up to 12 invasive *S*. Typhimurium ST313 isolates. We also included the reference strains *S*. Typhimurium SL1344 (ST19), *S*. Typhi Ty2 and *S*. Paratyphi A ATCC9150 for comparison.

#### (i) Uptake by J774 murine macrophages

**Figure 1:**
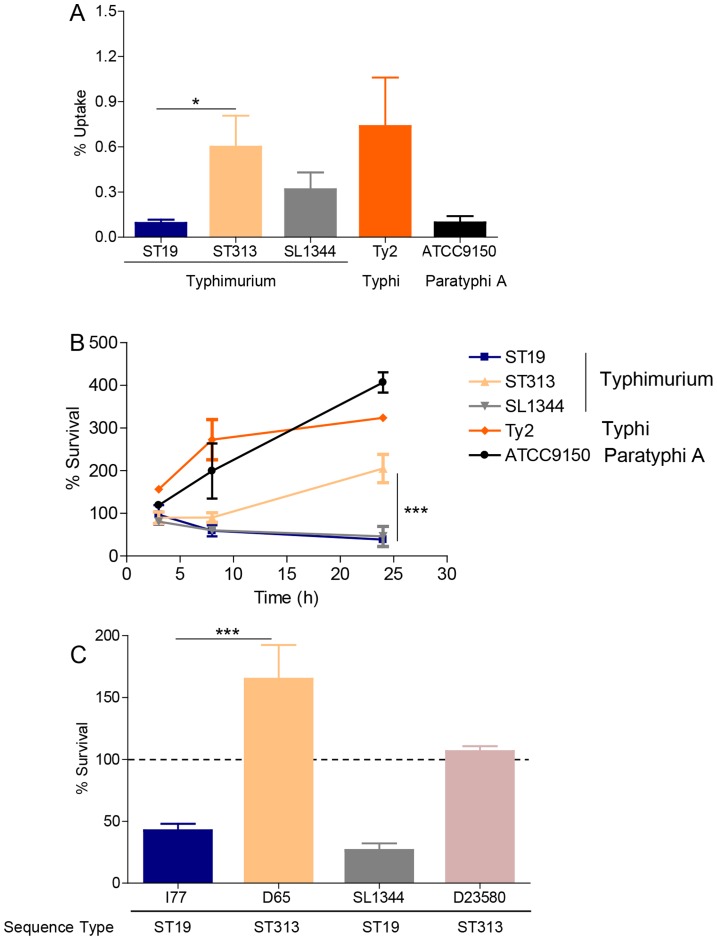
Interaction of *S*. Typhimurium ST19 and ST313 with macrophage cell lines. **(A)** J774 murine macrophages were infected with four strains of *S*. Typhimurium ST19 (I77, I41, S52 and I89), five strains of ST313 (D65, Q55, S11, S12 and A13), *S*. Typhimurium SL1344 (ST19), *S*. Typhi Ty2 and *S*. Paratyphi A ATCC9150. The ability of these strains to be phagocytosed by J774 macrophage cells was determined 30 min p.i. **(B)** U937 human macrophages were infected with four strains of *S*. Typhimurium ST19 (I77, I41, S52 and I89), five strains of ST313 (D65, Q55, S11, S12 and A13), *S*. Typhimurium SL1344 (ST19), *S*. Typhi Ty2 and *S*. Paratyphi A ATCC9150 in a gentamicin protection assay. Percent survival was expressed as the number of intracellular bacteria at 24 h p.i. divided by the number of intracellular bacteria at 0 h p.i. times 100. **(C)** *S*. Typhimurium I77 (ST19) and D65 (ST313) were used to infect human THP-1 cells. *S*. Typhimurium SL1344 (ST19) and *S*. Typhimurium D23580 (ST313) were used as controls for the assay. Percent survival was expressed as the number of intracellular bacteria at 24 h p.i. divided by the number of intracellular bacteria at 0 h p.i. times 100. Results are expressed as mean ± SD from at least 3 independent experiments. ** represents P <0.01.

To determine the differences in uptake of *S*. Typhimurium ST313 and ST19 strains, a phagocytosis assay was carried out using the J774 mouse macrophage cell line. After 30 min of infection, a significantly higher number of *S*. Typhimurium ST313 (n = 12 strains) were phagocytosed by J774 macrophage cells compared to *S*. Typhimurium ST19 strains (n = 4) (P <0.05; Fig. 1A).

#### (ii) Intracellular survival within human macrophage cell lines

To test the hypothesis that *S*. Typhimurium ST313 strains survive better within macrophages than *S*. Typhimurium ST19 strains, the U937 monocyte cell line was utilized in a gentamicin protection assay. After 24 h of incubation with U937 cells, we observed significantly more survival of *S*. Typhimurium ST313 strains (n = 5) than *S*. Typhimurium ST19 strains (n = 4) (P <0.001; Fig. 1B). Strains of the *S*. Typhimurium ST313 genotype not only survived but also replicated within the macrophages over the 24 h time period (as shown by greater than 100% survival). The control strains *S*. Typhi Ty2 and *S*. Paratyphi A ATCC9150 also replicated within the host cells (320%±1% and 390%±3%, mean ± standard deviation, respectively). In addition, we measured survival at 3 h and 8 h post-infection (p.i.) but no significant differences were observed between the *S*. Typhimurium ST19 and ST313 genotypes at these early time-points (Fig. 1B).

To confirm the observed intracellular survival results in other macrophage cell lines, THP-1 cells were infected with *S*. Typhimurium I77 (ST19) and D65 (ST313). At 24 h p.i., *S*. Typhimurium D65 (ST313) survived and replicated within THP-1 cells whereas *S*. Typhimurium I77 (ST19) was killed (160%±26% [mean ± sd] versus 47%±3%, respectively; P<0.001; Fig. 1C). We also tested survival of *S*. Typhimurium D23580; the ST313 sequenced strain from Malawi, and observed 107%±4% survival.

### *S*. Typhimurium ST313 strains survive and replicate within primary mouse and human macrophages

Since clear differences in survival were observed in macrophage cell lines, we also examined survival of *S*. Typhimurium ST19 and ST313 strains at 24 h p.i. within primary mouse peritoneal macrophages and human peripheral blood mononuclear cells (PBMCs) to determine if a similar phenotype would be observed. The reference strains *S*. Typhimurium SL1344 (ST19), *S*. Typhi Ty2 and *S*. Paratyphi A ATCC9150 were included for comparison.

#### (i) Survival within murine peritoneal macrophages

**Figure 2:**
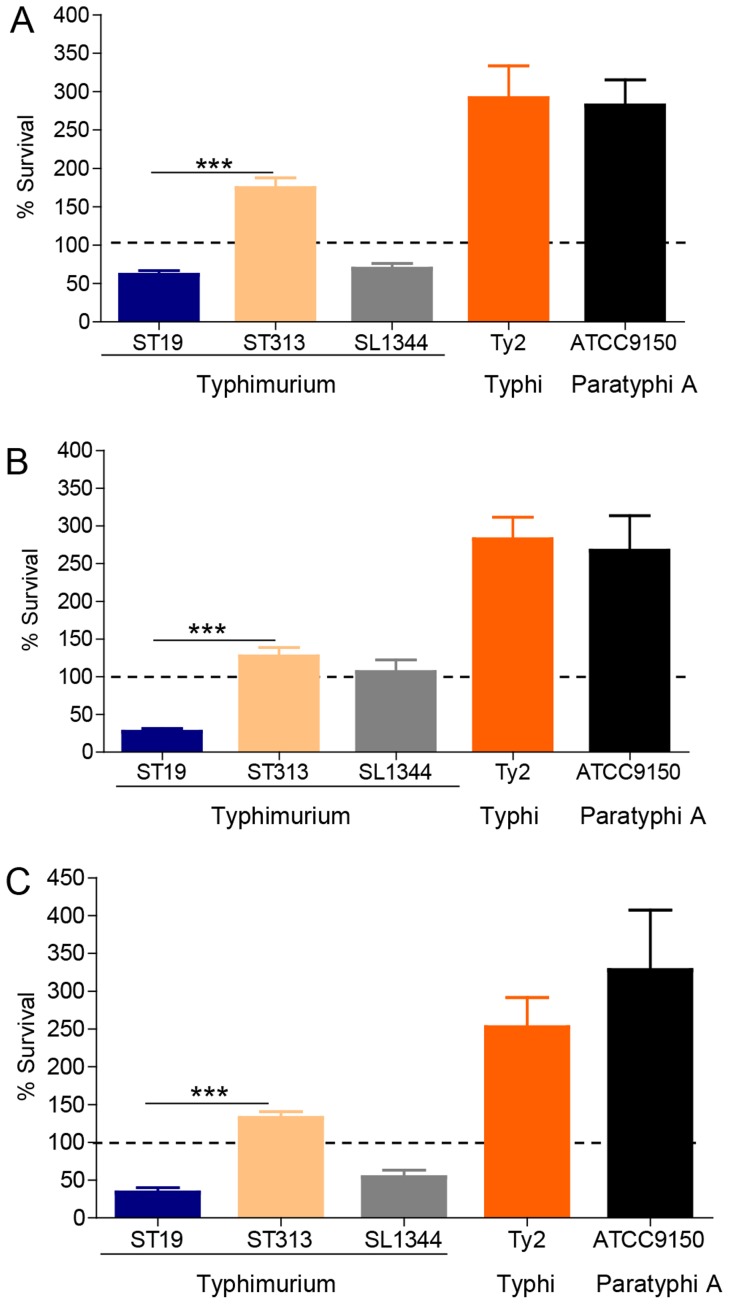
Survival of *S*. Typhimurium ST313 and ST19 within primary mouse and human macrophages. Peritoneal macrophages from BALB/c mice **(A)** or CD-1 mice **(B)** or PBMCs from human donors **(C)** were infected with four strains of *S*. Typhimurium ST19 (I77, I41, S52 and I89), five strains of ST313 (D65, Q55, S11, S12 and A13), *S*. Typhimurium SL1344 (ST19), *S*. Typhi Ty2 and *S*. Paratyphi A ATCC9150 at a MOI of 10∶1. Percent survival was expressed as the number of intracellular bacteria at 24 h p.i. divided by the number of intracellular bacteria at 0 h p.i. times 100. Results are expressed as mean ± SD from at least 3 independent experiments. *** represents P<0.001.

We determined that *S*. Typhimurium ST313 (n = 5) strains were able to replicate within peritoneal macrophages harvested from BALB/c mice whereas *S*. Typhimurium ST19 strains were killed (n = 4) (Fig. 2A). We observed a highly significant difference in percent survival between these two genotypes (P<0.001). Since BALB/c mice possess a mutated *Nramp1* allele which results in an inability of their macrophages to control *Salmonella* infections, we also tested survival within peritoneal macrophages from CD-1 outbred mice which possess the wild-type allele [23], [24]. Similar to that observed in BALB/c, there was a significant difference in percent survival within CD-1 peritoneal macrophages between *S*. Typhimurium ST19 and ST313 strains (P<0.001; Fig. 2B). In general, the overall percent survival of all strains tested in CD-1 peritoneal macrophages was lower than that observed for macrophages from BALB/c mice. For example, *S*. Typhimurium ST313 strains exhibited 175%±16% survival within BALB/c macrophages and only 130%±6% survival within CD-1 macrophages (P<0.001).

#### (ii) Survival within human peripheral blood mononuclear cells (PBMCs)

To test the ability of *S*. Typhimurium ST19 and ST313 strains to survive within human inflammatory cells, fresh human PBMCs from healthy volunteers were infected in a gentamicin protection assay. Human PBMCs efficiently killed the *S*. Typhimurium ST19 strains (n = 4). Only 37%±2% survival was observed with these strains after 24 h of incubation. In contrast, the *S*. Typhimurium ST313 strains (n = 5) survived and replicated within human PBMCs (140%±5% survival, P <0.001; Fig. 2C). Once again, *S*. Paratyphi ATCC1950 and *S*. Typhi Ty2 survived most efficiently (330±28% and 262%±19%, respectively) within these cells after 24 h of incubation (Fig. 2C).

### Visualization of *S*. Typhimurium D65 (ST313) and I77 (ST19) within human THP-1 cells

**Figure 3:**
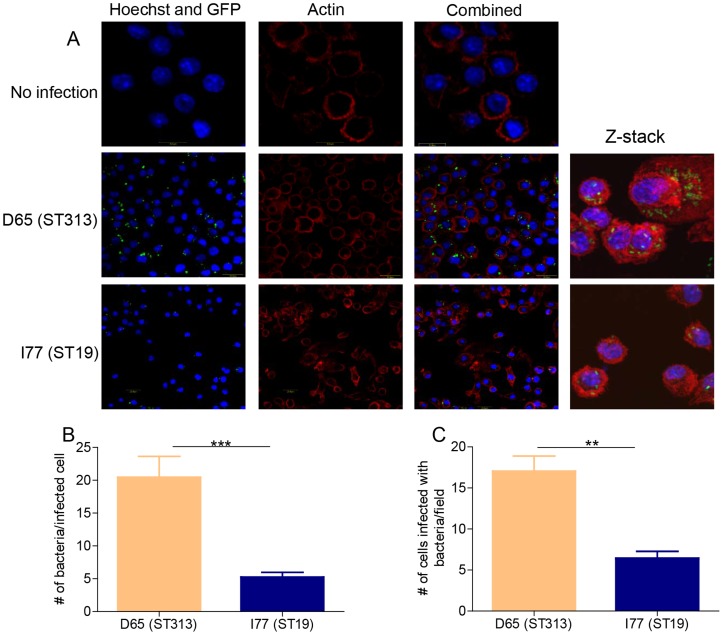
Confocal microscopy analysis of *S*. Typhimurium ST313 and ST19 within THP-1 cells. Human THP-1 cells were left uninfected or infected with GFP-expressing *S*. Typhimurium I77 (pGEN206) or *S*. Typhimurium D65 (pGEN206). **(A)** The cells were fixed 24 h p.i. and stained with Acti-stain phalloidin (Red) to stain for actin and Hoechst (Blue) to stain for the nucleus. Cells were viewed under a confocal microscope. Z-stack analysis was performed for the infected cells. At least six different regions of the slide for each condition were analyzed. **(B)** The number of bacteria per infected cell was determined by counting at least 12 cells in six different regions of the slide. **(C)** The number of cells infected with *S*. Typhimurium I77 (ST19) and D65 (ST313) per field was determined by viewing 12 different fields. ** represents P <0.01.

Confocal microscopy analysis of THP-1 cells infected with *gfp*-expressing *S*. Typhimurium D65 (ST313) and I77 (ST19) at 24 h p.i., revealed a larger number of intracellular *S*. Typhimurium D65 (pGEN206) cells as compared to *S*. Typhimurium I77 (pGEN206) (Fig. 3A). The number of bacteria per infected THP-1 cell was four-fold higher (∼20 bacteria/cell) in cells infected with *S*. Typhimurium D65 (ST313) compared to cells infected with *S*. Typhimurium I77 (ST19) (∼5 bacteria/cell) (P <0.001) (Fig. 3B). Furthermore, we observed greater than two-fold more infected THP-1 cells per field for *S*. Typhimurium D65 (ST313) compared to *S*. Typhimurium I77 (ST19) (P <0.01) (Fig. 3C).

### Host cell effects of infection with invasive *S*. Typhimurium

#### (i) THP-1 macrophages infected with the *S*. Typhimurium strain I77 (ST19) are more apoptotic than macrophages infected with *S*. Typhimurium D65 (ST313)

**Figure 4:**
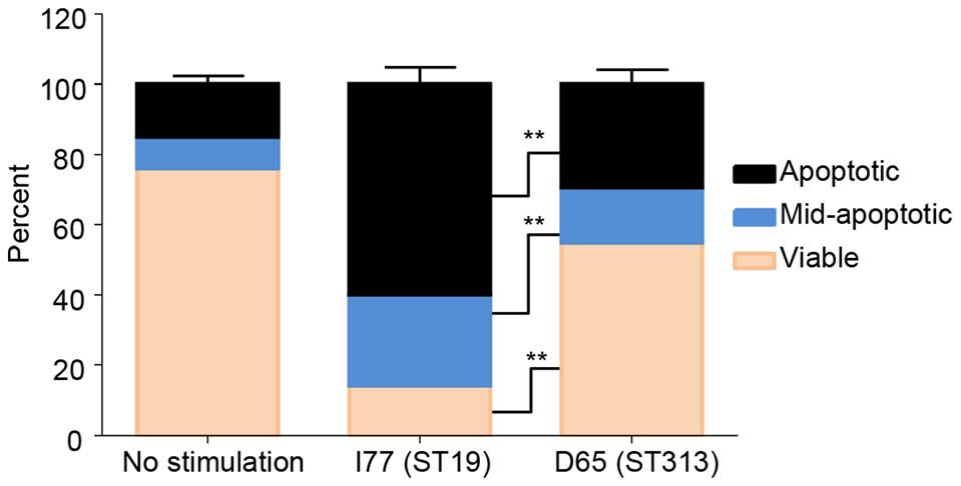
Toxicity assay to determine the effect of *S*. Typhimurium ST313 and ST19 on THP-1 macrophages. Human THP-1 cells were infected with *S*. Typhimurium I77 (ST19) or *S*. Typhimurium D65 (ST313) at a MOI of 10∶1 or left uninfected. Guava ViaCount was used to determine the number of viable, mid-apoptotic and apoptotic cells 24 h p.i. Results are expressed as mean ± SD from 3 independent experiments. ** represents P<0.01.

To determine the mechanism by which *S*. Typhimurium ST313 survive better than ST19 within macrophages, a Guava ViaCount toxicity assay was performed to quantitate the amount of apoptosis of THP-1 cells infected with strains of the *S*. Typhimurium ST19 and ST313 genotypes. At 24 h p.i., significantly more THP-1 cells infected with *S*. Typhimurium D65 (ST313) were viable compared to *S*. Typhimurium I77 (ST19) (P <0.01; Fig. 4). The majority of the cells infected with *S*. Typhimurium I77 were apoptotic (60±3%, mean ± sd) or mid-apoptotic (24±5%), compared to THP-1 cells infected with *S*. Typhimurium D65 (ST313) (P<0.01) (Fig. 4).

#### (ii) *S*. Typhimurium I77 (ST19) induces more proinflammatory cytokines compared to *S*. Typhimurium D65 (ST313)

**Figure 5:**
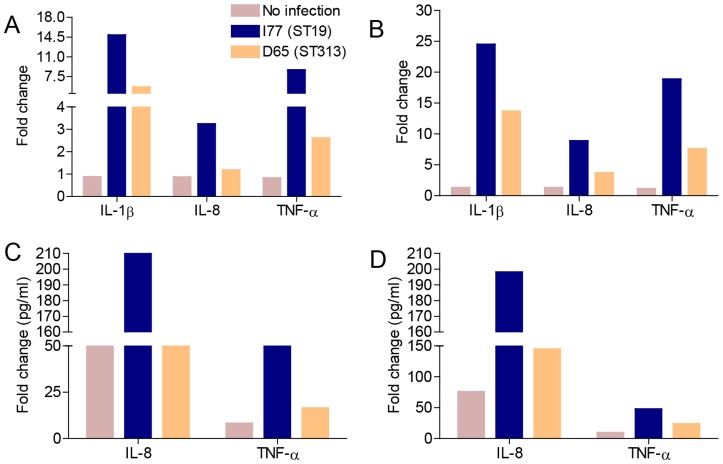
Cytokine expression of THP-1 cells infected with *S*. Typhimurium ST313 and ST19. Human THP-1 cells were infected with *S*. Typhimurium I77 (ST19), D65 (ST313) or left uninfected. **(A)** Total mRNA was isolated from the cells and expression profiles for different proinflammatory cytokines, IL-1β, IL-8 and TNF-α were analyzed by qRT-PCR at 3 h p.i. **(B)** IL-1β, IL-8 and TNF-α gene expression at 8 h p.i. (**C)** IL-8 and TNF-α protein expression analyzed at 3 h p.i. **(D)** IL-8 and TNF-α protein expression analyzed at 8 h p.i.

To further characterize the mechanism by which *S*. Typhimurium ST19 but not ST313 strains induce host cell death, proinflammatory cytokine mRNA profiles of THP-1 cells infected with *S*. Typhimurium I77 (ST19) and D65 (ST313) were determined by reverse transcriptase real-time PCR (RT-qPCR) at 3 and 8 h post-infection. At 3 h p.i., THP-1 cells infected with *S*. Typhimurium I77 (ST19) induced a 2.6-, 2.8- and 3.3-fold increase in IL-1β, IL-8 and TNF-α gene expression, respectively, in comparison to cells infected with *S*. Typhimurium D65 (ST313) (Fig. 5A). A 1.8-, 2.4- and 2.5-fold increase in IL-1β, IL-8 and TNF-α gene expression, respectively, was also observed at 8 h p.i. in cells infected with *S*. Typhimurium I77 (ST19) compared to *S*. Typhimurium D65 (ST313) (Fig. 5B). Proinflammatory cytokine protein production was also measured by ELISA in the supernatant of THP-1 cells infected with *S*. Typhimurium I77 (ST19) and *S*. Typhimurium D65 (ST313) and confirmed the RT-qPCR results. At 3 h p.i., there was a 1.4-fold increase in IL-8 and a 2-fold increase in TNF-α produced by cells infected with *S*. Typhimurium I77 (ST19) compared to *S*. Typhimurium D65 (ST313) (Fig. 5C), whereas at 8 h p.i. there was a 1.3-fold increase in IL-8 and 2.1-fold increase in TNF-α production by THP-1 cells infected with *S*. Typhimurium I77 (ST19) compared to *S*. Typhimurium D65 (ST313) (Fig. 5D). The gene and protein expression of several other cytokines including IFN-γ, IL-4, IL-6 and IL-10 were also analyzed, however, the levels of these cytokines were below the limit of detection.

#### (iii) *S*. Typhimurium I77 (ST19) enhances phosphorylation of p38 and p65 compared to *S*. Typhimurium D65 (ST313)

**Figure 6:**
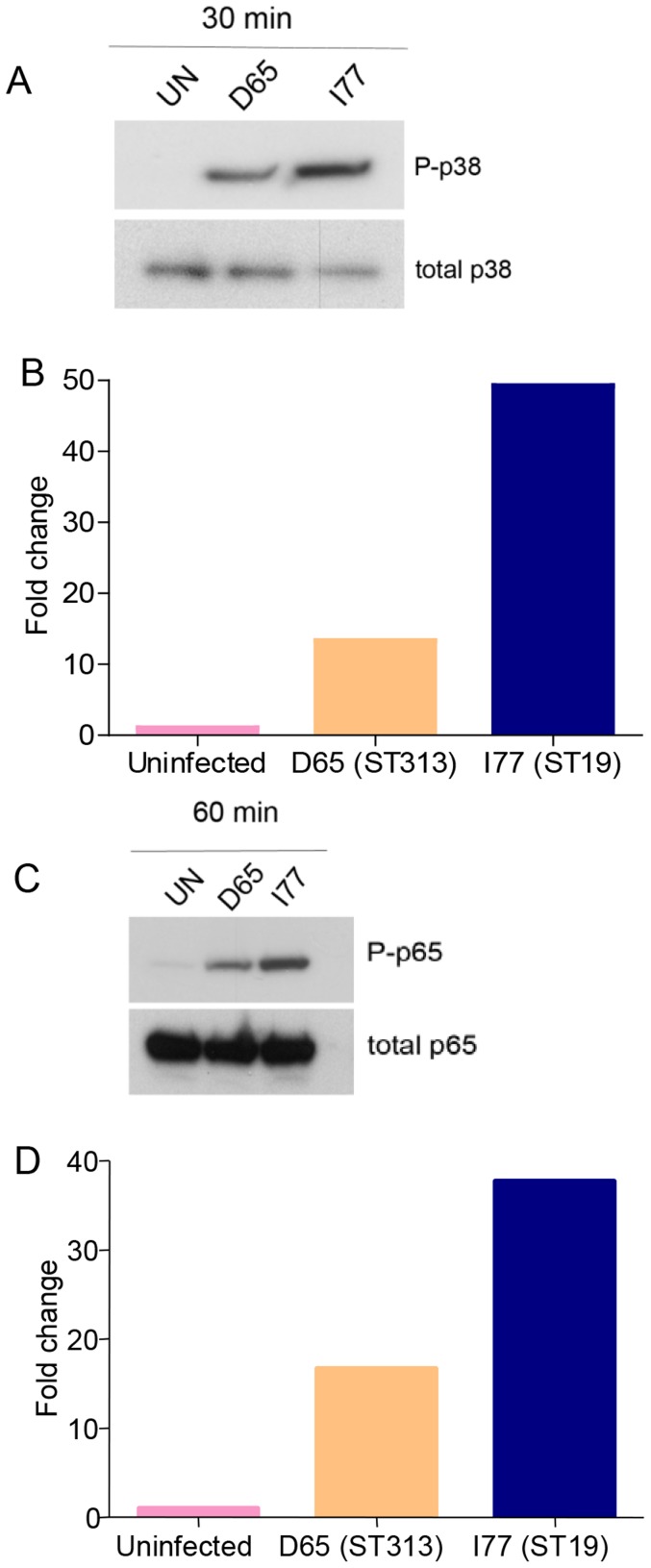
NFκB expression in THP-1 cells infected with *S*. Typhimurium ST313 and ST19. **(A)** Induction of phosphorylated p38 in THP-1 cells infected with *S*. Typhimurium D65 (ST313) or I77 (ST19) 30 min p.i. by Western blot analysis using polyclonal rabbit-anti-phosphorylated p38. **(B)** Densitometry quantification of P-p38 at 30 min p.i. of THP-1 cells by ImageJ software. **(C)** Induction of P-p65 in THP-1 cells infected with *S*. Typhimurium D65 (ST313) or I77 (ST19) at 60 min p.i. **(D)** Densitometry quantification of P-p65 in THP-1 cells by ImageJ software at 60 min p.i. with *S*. Typhimurium D65 (ST313) or I77 (ST19).

To investigate differences in activation of the innate immune system of *S*. Typhimurium ST19 and ST313 strains, we examined the phosphorylation of p38, a mitogen-activated protein kinase (MAPK) that responds to stress stimuli, and phosphorylation of p65, a transcription factor that is required for NF-κB activation and function. Both the NF-kB and MAPK pathways are coordinately activated downstream of the Toll-Like Receptors (TLRs) including TLR5 [25]. THP-1 cells were infected with *S*. Typhimurium I77 (ST19), *S*. Typhimurium D65 (ST313) or left uninfected. As early as 30 min p.i., enhanced phosphorylation of p38 was observed in cells infected with *S*. Typhimurium I77 (ST19) compared to *S*. Typhimurium D65 (ST313) (Fig. 6A). We observed more than three-fold higher induction of phosphorylated p38 in THP-1 cells infected with *S*. Typhimurium I77 (ST19) compared to *S*. Typhimurium D65 (ST313) (P <0.01) (Fig. 6B). Similarly at 60 min p.i., THP-1 cells infected with *S*. Typhimurium I77 (ST19) induced significantly more phosphorylated p65 than *S*. Typhimurium D65 (ST313) (P <0.01) (Fig. 6C and D).

### Contribution of flagellin to *S*. Typhimurium ST19 and ST313

**Figure 7:**
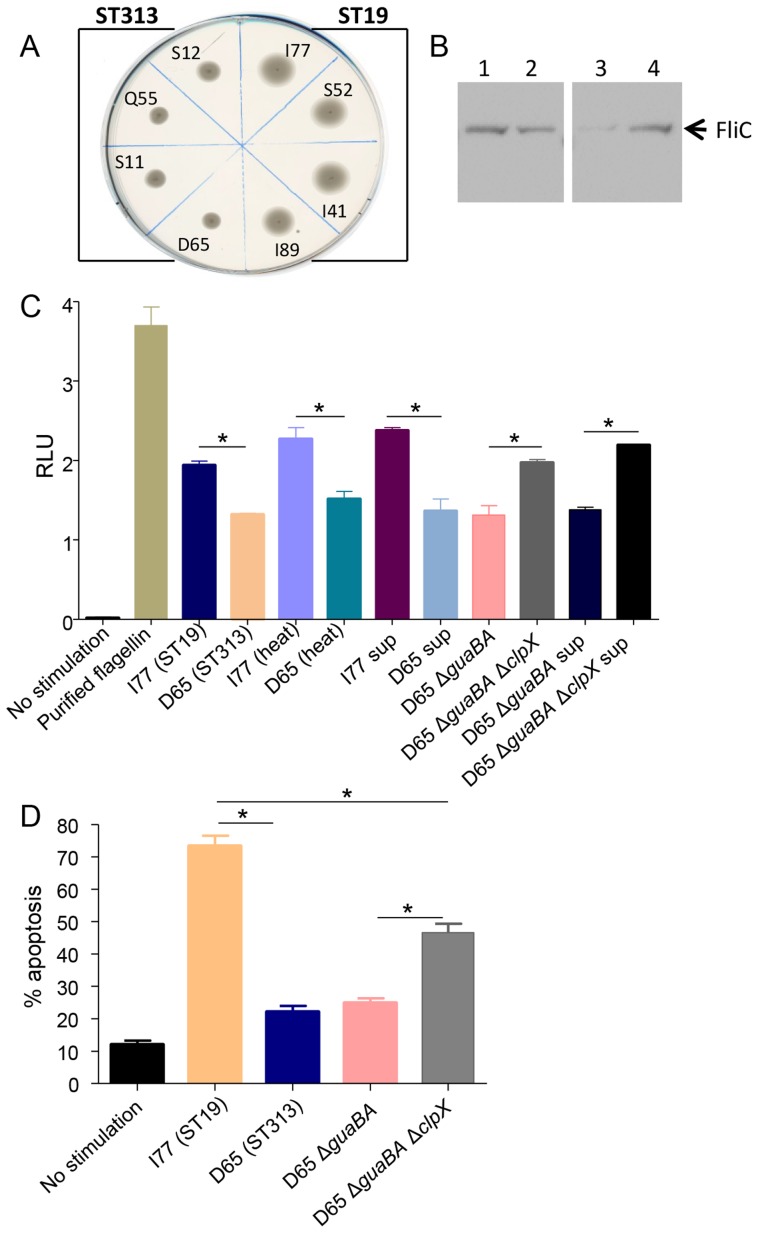
Flagella expression in *S*. Typhimurium ST19 and ST313 strains. **(A)** Motility assay using *S*. Typhimurium ST313 (D65, Q55, S11, S12) and *S*. Typhimurium ST19 (I77, S52, I41, I89). **(B)** Bacterial supernatant of *S*. Typhimurium (1) I77 (ST19), (2) D65 (ST313), (3) D65 Δ*guaBA* and (4) D65 Δ*guaBA* Δ*clpX* were analyzed by Western blot using a monoclonal antibody against *S*. Typhimurium flagellin. **(C)** Human HEK293 cells stably transfected with a firefly luciferase reporter gene under control of the NF-κB promoter were infected in triplicate with live or heat-killed wild-type *S.* Typhimurium I77 (ST19), D65 (ST313), D65 Δ*guaBA* and D65 Δ*guaBA* Δ*clpX* which overexpresses flagellin. Culture supernatants from all the strains were also used to activate NF-κB expression. HEK293 cells were harvested at 4 h after infection and luciferase activity (RLU) determined. Luciferase activity from infected cells is expressed relative to that obtained from uninfected cells. **(D)** Cytotoxicity assay on THP-1 cells infected with *S.* Typhimurium I77 (ST19), D65 (ST313), and D65 Δ*guaBA* and D65 Δ*guaBA* Δ*clpX* at 24 h p.i. * represents P<0.05.

In order to determine if the difference in inflammation and cell death elicited by *S*. Typhimurium ST19 and ST313 strains could be due to a difference in Pathogen-Associated Molecular Patterns (PAMPs), we examined flagellin production. We first tested motility of 4 ST19 and 16 ST313 *S*. Typhimurium strains using motility agar. The *S*. Typhimurium ST19 strains showed significantly larger zones of motility compared to *S*. Typhimurium ST313 strains (24 mm±2 mm versus 17 mm±3 mm, respectively; P<0.001, Student's t-test, two-tailed). Fig. 7A shows a representative motility assay for 4 *S*. Typhimurium ST313 and ST19 strains. To determine whether the difference in motility could be due to differences in the quantity of flagellin produced by each genotype, we performed Western blot analysis using a monoclonal antibody against *S*. Typhimurium Phase 1 flagellin (FliC-i). As shown in Fig. 7B, larger amounts of FliC-i were produced by *S*. Typhimurium I77 (ST19) compared to *S*. Typhimurium D65 (ST313).

Since flagellin activates the innate immune system by direct interaction with TLR5, we assessed whether TLR5 activity was enhanced for *S*. Typhimurium I77 (ST19) compared to *S*. Typhimurium D65 (ST313). We characterized the expression of flagella by these strains in a TLR5-dependent NF-κB induction assay. We observed a significant increase in NF-κB induction when HEK293-Luc cells were infected with *S*. Typhimurium I77 (ST19) compared to *S*. Typhimurium D65 (ST313) (P<0.05; Fig. 7C). Significant differences were observed when live or heat-killed bacteria or supernatant was used to infect HEK-293-Luc cells. To determine whether NF-κB induction by *S*. Typhimurium D65 could be enhanced by increasing flagella production we tested a recombinant *S*. Typhimurium D65 strain that we have previously constructed and which hyperexpresses flagella. The ClpPX protease normally degrades the master flagella regulator FlhDC and when it is absent, strains become hyperflagellated [19], [26], [27]. We used *S*. Typhimurium D65 Δ*guaBA* Δ*clpX* which expresses more FliC than the parental strain *S*. Typhimurium D65 Δ*guaBA* (Fig. 7B). The levels of NF-κB induced by cells infected with *S*. Typhimurium D65 Δ*guaBA* Δ*clpX* whole bacteria or supernatant were comparable to levels induced by *S*. Typhimurium I77 (ST19) (Fig. 7C). This work showed that we could hyperexpress *S*. Typhimurium ST313 flagellin and therefore TLR5 activation to the same levels as *S*. Typhimurium ST19 flagellin.

To determine whether the observed differences in cytotoxicity induced by *S*. Typhimurium I77 and D65 in THP-1 cells could be due to differences in flagellin expression, we measured cytotoxicity due to *S*. Typhimurium D65 Δ*guaBA* Δ*clpX.* Interestingly, overexpression of flagella in the *S*. Typhimurium D65 background (*S*. Typhimurium D65 Δ*guaBA* Δ*clpX*) led to greater than a two-fold increase in cytotoxicity (P<0.05) compared to *S*. Typhimurium D65 Δ*guaBA*. However, despite activating as much TLR5 as *S*. Typhimurium I77, *S*. Typhimurium D65 Δ*guaBA* Δ*clpX* still produced significantly less apoptosis than *S*. Typhimurium I77 (P<0.05) (Fig. 7D).

## Discussion

Our findings are consistent with earlier studies that showed that *S*. Typhi survives better within human macrophages compared to *S*. Typhimurium [28], [29]. There are conflicting reports about survival of *S*. Typhi and *S*. Typhimurium within macrophages in the literature; some investigators have found that *S*. Typhimurium shows higher survival than *S*. Typhi and some groups have reported the reverse [30], [31]. These discrepancies may be due to differences in methodology. In this study, we grew *Salmonella* in bacteriological medium containing 0.3 M NaCl and harvested at an OD_600_ of 1.5. However, Spano and Galan [32] also used media containing 0.3 M NaCl and harvested bacteria at an OD_600_ of 0.9, but observed reduced survival of *S*. Typhi in bone-marrow derived macrophages compared to *S*. Typhimurium.

Notably percent survival of all *Salmonella* strains tested in BALB/c macrophages was higher than that observed for CD-1 macrophages. This could be due to the presence of a nonfunctional allele of the natural resistance associated macrophage protein 1 (*Nramp1*) gene in BALB/c mice [33]. This gene encodes a transmembrane protein that transports divalent cations to the lysosome, a mechanism important for eliminating bacteria from host cells [23], [34]. As such, BALB/c mice are highly susceptible to *Salmonella* infection whereas CD-1 mice, which possess wild-type Nramp1, are highly resistant. Throughout the course of our study, we observed that *S*. Typhimurium SL1344 showed the same phenotype as clinical *S*. Typhimurium ST19 strains from Mali except for the experiment measuring survival in peritoneal macrophages from CD-1 mice. The reason for this difference is unknown but it may reflect an artifact of adaptation of *S*. Typhimurium SL1344 to laboratory passage conditions.

A new putative virulence gene *st313-td* associated with macrophage survival was shown to be present in *S*. Typhimurium ST313 from Nigeria and Democratic Republic of Congo [35]. A mutant strain lacking *st313-td* displayed significantly decreased intracellular survival within macrophages compared to the wild-type strain at a late time-point of infection. Evidence suggests that *st313-td* plays a dual role in decreased uptake of bacteria during opsonophagocytosis but increased survival of the bacteria at late time-points [36]. The role of the *st313-td* gene in the *S*. Typhimurium ST313 clinical isolates from Mali remains to be studied.

*S*. Typhimurium has been shown to invade macrophages and induce a rapid cell death or persist within a phagocytic vacuole and establish a niche [37]. This early rapid cell death is dependent on *Salmonella* pathogenicity island (SPI)-1 and the host caspase-1 protein, and late-phase macrophage cell death is mainly dependent on SPI-2-encoded genes [38]. Early studies showed that *S*. Typhi induced significantly less apoptosis than *S*. Typhimurium SL1344 in RAW264.7 macrophages and bone-marrow derived macrophages [39]. Our data shows that *S*. Typhimurium D65 (ST313) is similar to *S*. Typhi *in vitro* in that it causes reduced host cell death compared to *S*. Typhimurium ST19.

The molecular mechanisms by which *S*. Typhimurium initiates killing of macrophages are still being elucidated. Nevertheless, there are definitive data demonstrating caspase-1 dependent inflammasome activation and cytotoxicity following cytosolic recognition of bacterial constituents by macrophage NLRP3 and NLRC4 receptors, a process known as pyroptosis [40]. Caspase-1, also known as IL-1β converting enzyme, causes maturation and secretion of intracellular pro-IL-1β. Coupled with the higher levels of apoptosis, our finding that higher levels of IL-1β can be found in the supernatants of macrophages infected with *S*. Typhimurium ST19 relative to ST313 indirectly supports the hypothesis that ST313 *S*. Typhimurium strains activate the pyroptotic pathway less vigorously than ST19.

A study analyzing the transcriptional response in whole blood of HIV-positive individuals infected with iNTS in Malawi observed an attenuation of NF-κB mediated inflammation and innate immune signaling compared to HIV-positive individuals infected with other bacterial pathogens [41]. Based on our findings, we postulate that this downregulation of innate immune signaling is not specific to HIV-infected individuals infected with iNTS but can be attributed to the *S*. Typhimurium ST313 pathogen. Indeed, we show that one of the mechanisms which these bacteria use to reduce inflammation is by decreasing flagellin production.

Taken together, our data suggests that *S*. Typhimurium ST19 strains hyperexpress flagellin which activates the innate immune system extracellularly via TLR5. TLR5 activation subsequently leads to increased production of pro-inflammatory cytokines and inflammation. Flagellin is also recognized by the inflammasome intracellularly which ultimately leads to caspase-1 activation and cell death. In contrast, *S*. Typhimurium ST313 strains produce a ‘silent’ infection in macrophages similar to that observed for *S*. Typhi and *S*. Paratyphi A. These pathogens are phagocytosed by macrophages and then replicate to high levels and in the process, cause minimal cell damage or inflammation. A similar phenomenon has recently been described for *S*. Dublin blood isolates. Yim *et al* [42] found that 4 out of 7 *S*. Dublin blood isolates were aflagellate compared to 3 isolates from other sources that were flagellated. They further showed that the aflagellate isolates elicit less inflammation. In contrast, we show that *S*. Typhimurium ST313 strains still produce flagella albeit at a lower level than *S*. Typhimurium ST19 strains. The genetic basis of reduced flagella production by *S*. Typhimurium ST313 is not known.

This phenomenon of decreased inflammation and reduced apoptosis is, however, not entirely due to downregulation of flagellin expression. Our hyper-expressing ST313 flagellin strain does not induce apoptosis to the levels triggered by *S*. Typhimurium I77 (ST19), suggesting that other factors might be involved in this phenotype. Furthermore, we observed differences in survival between the *S*. Typhimurium ST19 and ST313 strains in mouse peritoneal macrophages which do not express TLR5 [43] but do express cytosolic receptors that can bind flagellin and initiate apoptosis. Since flagellin is the major activator of TLR5, this further suggests that factors other than extracellular activation of TLRs are important for survival within macrophages.

Several groups have investigated the co-infection of non-typhoidal *Salmonella* (NTS) with malaria parasites or SIV in laboratory models to understand how individuals with malaria or HIV are predisposed to NTS infection [44], [45]. However, very few groups have used invasive *S*. Typhimurium ST313 strains isolated from febrile patients in sub-Saharan Africa for their studies. More work should be done to understand how these particular pathogens take advantage of certain co-morbidities.

In summary, invasive *S*. Typhimurium ST313 clinical isolates from Mali have enhanced survival within human and murine macrophages while causing minimal inflammatory responses compared to *S*. Typhimurium ST19. This may explain why *S*. Typhimurium ST313 strains are more commonly isolated from blood of febrile patients in sub-Saharan Africa than *S*. Typhimurium ST19 strains. We also provide evidence that indicates that part of the inflammatory response elicited by *S*. Typhimurium ST19 strains is due to increased flagella production. However, our data also suggests other factors can contribute to inflammation of *S*. Typhimurium ST19 and which are absent in *S*. Typhimurium ST313.
